# Rethinking autism: implications of sensory and movement differences for understanding and support

**DOI:** 10.3389/fnint.2012.00124

**Published:** 2013-01-28

**Authors:** Anne M. Donnellan, David A. Hill, Martha R. Leary

**Affiliations:** ^1^School of Leadership and Education Sciences, University of San DiegoSan Diego, CA, USA; ^2^Tri-County Regional School BoardYarmouth, NS, Canada; ^3^Private PracticeHalifax, NS, Canada

**Keywords:** autism, autism: sensory-movement differences, autism: sensory-motor difficulties, autism: neurological implications, autism: movement perspective

## Abstract

For decades autism has been defined as a triad of deficits in social interaction, communication, and imaginative play. Though there is now broad acknowledgment of the neurological basis of autism, there is little attention paid to the contribution of such neurological differences to a person's development and functioning. Communication, relationship, and participation require neurological systems to coordinate and synchronize the organization and regulation of sensory information and movement. Developmental differences in these abilities are likely to result in differences in the way a person behaves and expresses intention and meaning. The present paper shares our emerging awareness that people may struggle with difficulties that are not immediately evident to an outsider. This paper explores the symptoms of sensory and movement differences and the possible implications for autistic people. It provides a review of the history and literature that describes the neurological basis for many of the socalled behavioral differences that people experience. The paper emphasizes the importance of our acknowledgment that a social interpretation of differences in behavior, relationship, and communication can lead us far away from the lived experience of individuals with the autism label and those who support them. We suggest alternative ways to address the challenges faced by people with autism.

## Introduction

I was intensely preoccupied with the movement of the spinning coin or lid and I saw nothing and heard nothing. I did it because it shut out sound that hurt my ears. No sound intruded on my fixation. It was like being deaf. Even a sudden noise didn't startle me out of my world.

(Grandin, 1992a,b,c)

People with autism often move their bodies in ways that are unfamiliar to us. Some people rock, repeatedly touch an object, jump, and finger posture while other people come to a standstill in a doorway, sit until cued to move or turn away when someone beckons. As professionals trained to see these as *autistic behaviors*, most of us have interpreted such movements as both volitional and meaningless; or as communicative acts signaling avoidance of interaction and evidence of diminished cognitive capacity; or as some combination of these, often to be targeted for reduction. We have taken a socially constructed interpretation of what we see and have built a “theory” of autism.

This paper challenges the traditional definitions of autism that give primacy to a triad of deficits in social interaction, communication, and imaginative play (Wing, 1981; *DSM-IV- TR* American Psychiatric Association, 2000). The approach is both widely known and essentially unchallenged despite broad acknowledgment that autism is a condition that reflects some differences in a person's neurology. Typically, the neurological implications have not become part of the description. Over the past two decades, however, researchers and self-advocates have begun to rethink this socially defined focus. They express concern that children and adults with the autism label may be challenged by unrecognized and significant sensory and movement differences (e.g., (Williams, 1992; Hill and Leary, 1993; Donnellan and Leary, 1995; Bristol et al., 1996; Leary and Hill, 1996; Donnellan, 1999; Filipek et al., 2000; Sullivan, 2002; Dhossche, 2004; Bluestone, 2005; Nayate et al., 2005; Endow, 2006; Jansiewicz et al., 2006; Mostofsky et al., 2006; Leekam et al., 2007; Markram et al., 2007; Tomchek and Dunn, 2007; Gernsbacher et al., 2008; Goldman et al., 2009).

Researchers and others describe these differences using a variety of terms such as: motor problems, sensory-integration problems, inertia, sensory overload, apraxia, dyspraxia, echolalia, mutism, behavior disorder, catatonia, or clumsiness. To reflect the range and complexity of sensory perception and movement related phenomena, we use the term “sensory and movement differences” as it encompasses the dynamic interaction of sensation and movement (Gibson, 1979; Thelen and Smith, 1994) while acknowledging that many differences are merely part of the richness of human diversity.

Behavior is highly interpretable. Some behaviors may be communicative; some may be volitional (Donnellan et al., 1984). Some behaviors, however, may not be intentional. Rather, observed behaviors may be artifacts of the difficulties a person may be having in organizing and regulating sensation and movement. Still others may be subtle signals of the desire for relationship or expressions of meaning. Therapeutic and intervention-based approaches, designed to address perceived and identified challenging and problematic behaviors of individuals with autism, tend to oversimplify the complex nature of human interactions in an attempt to delineate and manipulate variables contributing to and sustaining particular behaviors.

As we have *professionalized* interactions with people with autism, we have trained professionals, parents, and others to interpret what happens in terms of simple, binary views of behavior (i.e., good/bad or positive/negative), and to see behaviors as controlled by immediate, situational antecedents, and consequences. When we focus on these socially constructed expectations for behavior and communication in our fast-paced, super-technological world, we miss opportunities to know and understand people who may experience their existence and interactions in very different ways. Behaviors may not be what they seem (Donnellan et al., 2006; Robledo et al., 2012).

Our interest in the topic of sensory and movement differences has grown from reports by many self-advocates with the autism label and their caregivers that disturbances of sensation and movement are a constant concern, frequently constraining ability to communicate, relate to others and participate in life (e.g., Barron and Barron, 1992; Strandt-Conroy, 1999; Rubin et al., 2001; Robledo et al., 2012). Organizing and regulating sensory information and movement in order to participate in social relationships may be frustrating for people with such differences. These differences can involve difficulties initiating and executing movements or difficulties with stopping, combining, and switching sensation and movement including speech, thought and emotion, (Hill and Leary, 1993; Donnellan and Leary, 1995; Donnellan et al., 2006) making social relationships and many other activities very challenging, even overwhelming.

Self-advocates also report that they lack sensation or feedback from their bodies and may feel physically unaware of their facial expressions, position in space and movements (e.g., Williams, 1996a,b, 2003; Blackman, 1999; Hale and Hale, 1999). Some experience the sights and sounds of their world as being painfully intense (Condon, 1985; Williams, 1992, 1996b; Markram et al., 2007). Extreme emotions can cause the individual to become stuck, unable to cease repetition of a movement. Self-confidence and reputation often suffer when others assume a person is repeating an action “on purpose.” Sean Barron (Barron and Barron, 1992, p. 181) wrote: “All I wanted was to be like the other kids my age. It felt as if I was weird and strange on the outside, but inside I was not like that. The inside person wanted to get out and break free of all the behaviors that I was a slave to and could not stop.” For many people, as for Sean, simple movements can lead to repetitions or perseveration, even when they want to stop the movement.

Our concern here is not to discard useful information already accumulated via a primarily socially defined approach to autism. Nor are we interested in enhancing a deficit-based approach to understanding autism, or in creating a new disability category. We do not propose to specify a cause of autism or a site of lesion or dysfunction within the central nervous system. Rather, we write to share our emerging awareness that people may struggle with difficulties that are not immediately evident to an outsider. That is, our experience of individuals with autism ought no longer to be assumed the same as their experience. Individuals with the autism label often describe experiences which are not immediately obvious to the rest of us but which may well-affect our understanding of their behavior. These experiences frequently fit the definition of sensory and movement differences. Sue Rubin (pers. communication, August 4, 2007) described her dilemma with intention and action: “When you said we could stay and asked dad to do the shopping for the Asperger's barbeque, my body relaxed and autism let me eat the melon.” And two other autistic adults had the following interaction about sensory and movement differences. Judy Endow (pers. communication via Facebook, January 25, 2009) described her experiences in relation to sensory and movement differences:

I think the fluidity of access to various places in the brain is dependent upon neurological movement between places. I'm no scientist, but have always been able to “see” this inside of me. Sometimes my speaking is hindered, other times my thinking and sometimes my physical movement. The hardest is when thinking is not working smoothly. When that happens I have to line up one thought at a time, like train cars. I like it much better when my thoughts do not have to be methodically lined up, but are more fluid with colors coming in and out and swirling into unique and beautiful patterns. (My thoughts are in pictures and sometimes moving colors.)

Phil Schwarz (pers. communication via Facebook, January 25, 2009) commented on Judy's description by using another analogy:

I think that processing bandwidth—what Judy calls “neurological movement between places”—is a critical factor in autism. I think that those of us who do learn to cope develop adaptations that allow more parsimonious use of the bandwidth available to us: love of sameness, or of patterns, or of predictability (so that we can apply the bandwidth we do have to “deviations” from the predicted or from the patterns). There is a coherent autistic aesthetic sensibility that is informed by this search for parsimony of bandwidth use, and for titration of excesses.

This paper explores some of the implications of sensory and movement differences in the development and experiences of individuals with the autism label. We note, of course, that some researchers and clinicians completely deny the possibility that individuals with autism might experience any problems with movement. Rimland (1993, p. 3), a psychologist long a proponent of a biological approach to autism, wrote:

It has been widely recognized for many decades that the vast majority of autistic persons are quite unimpaired with regard to their finger dexterity and gross motor capabilities. They have in fact often been described as especially dexterous and coordinated. The literature abounds with stories of young autistic children who can take apart and reassemble small mechanical devices, build towers of blocks and dominos higher than a normal adult can, assemble jigsaw puzzles and climb to dangerously high places without falling. The files of the Autism Research Institute contain over 17,000 questionnaires completed by the parents of autistic children. Finger dexterity is one question we've asked about since 1965. Most parents indicate that their children are average or above in the use of their hands. The idea that autism is, or typically involves, a “movement disorder” is simply ludicrous.

Likewise, Mulick et al. (1993), behavioral psychologists, stated unequivocally that clinical experience argues against any motor/movement difficulties, particularly voluntary control of movement in apraxia:

Scientific evidence for developmental apraxia in autism is lacking. Autistic youngsters are often characterized by better-developed [emphasis in original] motor skills than verbal skills, even real non-verbal problem solving talent … There is no research evidence at all to support the position that people with autism experience such global problems. The usual clinical finding, familiar to any psychologist who routinely works in this area, is that motor impairment and delay is much less common than communication disorder and delay.

(p. 274)

The common approach in autism pays scant attention to possible somatic difficulties resulting from neurological differences. Perhaps, this is a function of the dominance of psychology and psychiatry for the first 50 or more years of the autism story. Yet, some psychologists and psychiatrists did report movement differences and even catatonic symptoms in autism long before Rimland or Mulick et al. and others denied the existence of such evidence (e.g., Damasio and Maurer, 1978; Wing and Attwood, 1987). More recently, many researchers have noted the presence of impairments in basic motor skills: gait, posture, balance, speed, coordination (e.g., Ghaziuddin and Butler, 1998; Noterdaeme et al., 2002; Jansiewicz et al., 2006; Rinehart et al., 2006; Green et al., 2009; Mostofsky et al., 2009; Fournier et al., 2010). Fournier et al. (2010) in their meta-analysis of claims of motor differences in autism since 1981 write:

Based on our synthesis of the existing literature and comprehensive meta-analytic techniques, we conclude that ASD is associated with significant and widespread alterations in motor performance. Recent neuroanatomical and neurophysiologic studies implicate cortical and subcortical areas including the motor context, supplementary motor deficits in motor planning, sensorimotor integration, and motor execution …. Our current findings serve as the basis for tentatively arguing that motor deficits are a potential core feature of ASD, and that treatment of ASD should consider including interventions aimed at improving motor performances involved with motor coordination (i.e., gait and balance, arm functions, and movement planning).

(p. 1237)

Many neuroscientists now are stressing the significance and implications of motor and sensory difficulties in the development of children with autism. For example, Sutera et al. (2007) looked at 4-years-old who had been diagnosed at age two and received early intervention of various amounts and types. Of particular interest were the children who “lost” the diagnosis of autism by age four. Sutera et al., found that the best predictor of this outcome for very young children with autism is motor skill at age two. Mostofsky et al. (2007) noted this finding and addressed concerns about the exclusion of motor problems from the “core” features of autism in the *Diagnostic and Statistical Manual of Mental Disorders* (*DSM-IV-TR*; American Psychiatric Association, 2000) “… despite [an] abundance of literature suggesting otherwise.” A growing number of researchers and clinicians in a broad range of disciplines continue to stress the importance of studying motor function in autism because, as Rogers et al. (2003) reported, “Simple imitation skills were differentially impaired in young children with autism, and lack of social cooperation did not account for their poor performance p. 763). Mostofsky et al. (2007) reported, “Motor signs are highly quantifiable and reproducible and can serve as markers for deficits in parallel systems important for socialization and communication” (p. 2117). For example, children with autism are often described as lacking reciprocity. Esther Thelen (1941–2004), an innovative researcher of infant development, upon reviewing the issue of motor development in autism, asked: “How can you talk about “reciprocity” or lack thereof as a psychological phenomenon if the child has motor problems?” (pers. communication, 1997). In the course of development, if individuals move and respond in idiosyncratic ways from infancy, they will experience all interactions within a unique frame that most certainly differs from that which is called typical. The cumulative effect of such interactions will be one in which all aspects of relationships, including how to establish and maintain them, may be markedly skewed from the broader cultural consensus and expected rules of how relationships work^1^. Our experience and self-advocate reports have taught us that individuals with autism often are aware of their idiosyncrasies, may not be able to control them but do want communication, participation and relationship. In order to make this possible, we need to acknowledge and accommodate the differences so that communication, relationship, and participation can happen.

## Dynamic interactions of nervous system, body, and environment

As we have noted elsewhere (Donnellan et al., 2006), the writings of many authors interested in movement describe a unity of perception, action, emotion, and thought. Feldenkrais, a physicist, martial artist, and renowned movement innovator noted: “Our self-image consists of four components that are involved in every action: movement, sensation, feeling and thought” (Feldenkrais, 1972, p. 10). Likewise, in his fascinating book, *Awakenings*, Sacks (1990) wrote of the self-reports of his patients with post-encephalitic Parkinson's disease who temporarily “awoke” through the use of the drug L-Dopa. They all had been sick from the same disease, *Encephalitis lethargica*. The area of damage in their brains from the disease was clearly established. Nonetheless, each developed his or her own personalized version of movement disorder and many of their difficulties were unknown to the medical staff until they were able to speak. The variety of manifestations of symptoms encompassed difficulties with many hidden aspects of human experience: perception of the passing of time, interest in normal activities, fatigue, memory, and recurring thoughts. These complex phenomenon related to organization and regulation, now commonly recognized in other neurological disorders, require us to think about movement disorders beyond observable motor difficulties.

Thelen incorporated dynamic systems models in her innovative research on movement in child development (Thelen and Smith, 1994; Thelen, 1995). In this view, perceptions, movement, thoughts, and emotions can be linked together by having coincidentally (and possibly routinely) co-occurred. Experience may selectively reinforce them as a bundle. They can be unbundled or softly assembled as required by the context. The individual is always operating within an environment or context and, as the context changes, systems scan, adjust, and shift as necessary to meet new demands. These contextual shifts play a vital role in movement. Context comes together with in such a way as to allow the movement to emerge or not; a movement and, indeed, the person or persons are part of the context (Thelen and Smith, 1994). As Bateson (1972) told us years ago, context is far more than what is left when we take out the part we wish to study.

No single component is causal in determining the movement. As these are dynamic systems, the components are the context that determine the product. Thelen and Smith (1994, p. 73) further explained that “… even behaviors that look wired in or program-driven can be seen as dynamically emergent: behavior is assembled by the nature of the task, and opportunistically recruits the necessary and available organic components (which themselves have dynamic histories) and environmental support.” These may be actions, thoughts, words, memories, or sense experiences. Recall Proust, where the taste of a cookie released the hundreds of pages of *Remembrance of Things Past.*

Thelen's approach offers new ways to understand the inconsistent abilities and disabilities of individuals with the autism label. Speech is an example of dynamic behavior. Speech is not lost or gained; it emerges when all necessary components, recruited and appropriately regulated and organized, allow its production. Stress often makes speech difficult or even impossible. And stress need not be negative; excitement may also cause difficulties. Paradoxically, for some people with sensory and movement differences, stress also may help produce speech. While presenting with the authors at an Autism Society of America conference in July 1996, Arthur Shawlow, Nobel laureate and father of an adult son with autism, reported that his son could say a complete, and original, context-appropriate sentence about once every 8–10 years. He asked the audience, how many parents had similar experiences and at least 18 sets of parents raised their hands. They met and compared notes. Most of the labeled children of these individuals were able to speak under extreme, often negative, circumstances. Some had only spoken once or twice in a lifetime.

Reports of this kind are not unusual in the sensory and movement differences literature, among the autism community or our own 100+ years of combined experience with children and adults with the autism label. More common are phenomena such as echolalia, mutism, speech uttered only under unique circumstances, e.g., speaking what they have written. In the dynamic system model the notion of emergence begins to give us a way to understand and perhaps support people with these differences. Robledo et al. (2012) report on 40 h of interviews with adults with autism who experienced such symptoms and more. The interviews had to be adjusted to the specialized needs of the interviewees. Several could only answer written questions sent in advance; others if they were on the phone and in a warm bath. Likewise, the autistic people in Robledo and Donnellan (2008) each had personalized supports to enable them to participate in the interviews. We refer to these specialized arrangements as accommodations after Luria (1932) and Sacks (1990). We define accommodations as adjustments or adaptations of an interaction, a task, situation, or the environment that assist a person to temporarily get around difficulties organizing and regulating sensory information or movement (for example, see Donnellan et al., 2006).

## Learning from neurological symptoms in other sensory and movement disorders

In our review of the history of movement differences we found early descriptions of catatonia in the work of Kahlbaum (1874/1973) which seemed startlingly familiar (see Hill and Leary, 1993; Donnellan and Leary, 1995; Starkstein et al., 1995; Leary and Hill, 1996). In the nineteenth century there was no clear distinction between neurological and psychiatric symptoms. As the two fields diverged in the early twentieth century, however, some conditions gravitated into one or the other. Catatonia is presently defined as a characteristic of certain kinds of schizophrenia, though many have argued over the years for a more neurological view of the disorder (Abrams and Taylor, 1976; Rogers, 1992). The discussion of where to place catatonia and catatonic symptoms is once again topical because of the plan to update the *Diagnostic and Statistical Manual* of the APA. Some, in fact, are arguing for the inclusion of catatonia as a separate diagnostic category or under “movement disturbances” (Taylor and Fink, 2003; Fink and Taylor, 2006; Penland et al., 2006; Caroff and Ungvari, 2007). Irrespective of that discussion, it is useful to look at the symptoms described by Kahlbaum and other early and recent authors as these may illuminate the symptoms seen in individuals with autism and other developmental disabilities.

In Table 1, the characteristic features and symptoms on the left side of the table are borrowed from descriptors specific to several kinds of movement disorders (Kahlbaum, 1874/1973; Fink and Taylor, 2003, 2006; Taylor et al., 2005; Caroff and Ungvari, 2007; The Movement Disorder Society, 2010). The list of movement disorders symptoms is not in any particular order or hierarchy; rather, symptoms are listed randomly as taken from the above literature sources. The intent here is to show the scope of symptoms by feature that may account for certain behaviors seen in autism. Examples of behaviors listed on the right side of Table 1 appear there because they have been discussed in a previously published review of the autism literature and movement disturbances (Leary and Hill, 1996). The majority of these have also been documented and observed throughout many years of clinical practice with a large number of individuals with autism across the life span.

**Table 1:** **Characteristic features of substantial movement disturbances and evidence of possible overlap of symptoms in autism**.

**Movement disturbance feature**	**Symptoms evidence in autism**
Repetitive motor actions	e.g., Tapping, touching, grimacing
Rhythmical, cyclical movements	e.g., Rocking, shrugging, squinting, pouting
Lack of initiation	Requires prompts and cues to perform
Difficulty imitating others' actions	Both immediate and delayed motor imitation difficulties
Echophenomena	Mimesis; elaborate copying of others' actions—verbal and/or motor
Immobility	Remains fixed and inert in position and posture for extended time periods
Withdrawal	Isolates self away from focal activity and others
Grimacing	Facial/oral-motor movements
Stereotypies	Repetitive movements of the hands, limbs, extemities, and whole body
Aversion	Of eye gaze and attention to others
Negativism	Oppositional actions elicited with passive movement and overall behavior
Automatic obedience; suggestibility	Extreme compliance in response to verbal suggestion and environmental cues
Rigidity	Muscles rigid to passive movement
Bradykinesia	Slowness of movements, feebleness
Tremor	Essential, intentional, rest, postural, etc.
Forced grasping	Of another's hands, wrists, etc., or items in the environment
Akinesia	Marked absence of action and movements
Akathisia	Motor restlessness, moves about but not goal-directed
Ataxia	Loss of coordination in motor action execution
Perseveration	Motor or other repeated behavior after being elicited an initial stimulus
Ambitendency	Appears “stuck” in indecisive, hesitant movements
Tics	Motor and/or verbal
Obstruction; blocking	Incomplete movement toward a goal—“gets stuck” en route to goal
Difficulty with stopping, cessation of movement	Will continue movements unless redirected or stopped by an external means
Mannerisms	Uses intact and entire motor action sequences out of context, e.g., salutes
Waxy flexibility	Automatic ease and compliance with assuming unusual postures for extended time
Ballismus	Violent, rapid and apparently involuntary actions and movements
Choreiform movements	Rapid and apparently involuntary traveling and “dancing” ripples of movement
Catalepsy (posturing)	Maintains seemingly uncomfortable and imposed postures for extended time
Atheloid movements	Slow, writhing movements and actions
Spasms	Muscular spasms of varying durations affecting muscle groups
Dystonias	Sustained torsion due to muscle contractions in varied muscle groups
Impulsivity	Actions and movements triggered suddenly
Self-injury, mutilation	Disturbing and persistent attempts to inflict pain on self
Excitement; frenzy	Marked episodes of extreme amounts of activity for extended time
Aggression, destruction	Unprecipitated violent actions directed to others and the environment
Stupor	Prolonged period of total immobility, lack of responsiveness and mutism
Rituals	Object-related actions on objects as part of a routine, repeated event
Motility changes	e.g., Toe walking, skipping, hopping
Changes in speech behavior	e.g., Mutism; question repetition; echolalia; verbigeration; logorrhoea; foreign accent; changes in prosody; difficulty modulating volume
Automatic changes	Changes in typical autonomic functions, e.g., heart rate, perspiration, breathing, core body temperature

Leary and Hill (1996) analyzed the literature on symptoms associated with established movement disorders and those associated with autism. The greatest difference among these disabilities was the interpretation of the symptoms. In Tourette syndrome, Parkinson's disorder and catatonia, there was a neurological interpretation of symptoms. A social rather than a neurological interpretation was applied if the person had a label of autism. That which is called a “tic” in a person with Tourette syndrome is most often assumed to be a “behavior” (and often a conscious choice) in a person with autism. For symptoms interpreted through a neurological lens, individuals tend to be appropriately supported. In autism, symptoms are viewed frequently as behaviors to be reduced or eliminated often with a negative intervention and results. Table 2 illustrates descriptions given to similar behaviors dependent on a person's diagnosis.

**Table 2:** **Differences in descriptions of behavior**.

**Neurological terms**	**Social interpretation of behavior**
Akinesia	Non-compliance, social indifference
Festination	Behavior excess, careless
Bradykinesia	Lazy, slow
Bradyphrenia	Mental retardation
Tics	Aberrant behavior
Obsessional/adventitious behaviors	Autistic behavior, “stims”

The sensory and movement differences reported by and observed in individuals with autism may have a significant impact on their and our ability to relate and participate in social interactions. A neurological view of symptoms possibly affecting autistic individuals will help us to understand further the nature of differences experienced by these individuals. While the psychological impact is very real as experienced first-hand by participants in such interactions, it is useful to suspend social interpretations of the symptoms so as not to mistakenly ascribe intent and volition to individuals whose behavior may be contrary to what really is intended and able to be communicated.

Detailed personal descriptions of movement and sensory differences found in other disabilities have given us some additional insight as to what it may be like for a person to deal with various symptoms such as compelling impulses, a loss of conscious control, lack of initiation, akinetic moments, and unusual ways of being in the world (e.g., McGoon, 1994). Frequently, the person has both the challenge of the movement difference and burden of blame and misunderstanding. In the Robledo et al. (2012) research it was often necessary to use vignettes from people with other sensory movement differences to enable the autistic interviewees to recognize their own experience. Most expressed gratitude for the opportunity to learn about movement differences as they often had blamed themselves for their behavior and all thought they were alone in having these difficulties.

## Implications of sensory and movement differences for understanding people labeled with autism

A different kind of science.

Woe to that science whose methods are developed in advance of its problems, so that the experimenter can see only those phases of a problem for which a method is already at hand.

(Murphy, 1939, p. 114)

We have stressed the neurological aspects of what are commonly thought of as autistic characteristics and behavior problems. We do not intend, however, to either suggest a whole new category of disabilities in autism or to eliminate the psychological aspects. The issues here are similar to the challenges faced by those interested in Tourette syndrome. The syndrome was elucidated before the fields of neurology and psychiatry diverged (Gilles de la Tourette, 1885). For many years, psychiatry dominated the discussion and the treatment. In the past few decades, there has been a far greater emphasis on the neurology of the disorder. Yet, it is clear that it is not possible to separate the neurological from the psychological in a living human being. As Sacks (1989) suggests, there is need for a different kind of science that views the individual as a whole person, mind and body. This shift has begun in Tourette syndrome. In addition, dynamic systems models of development suggest an emphasis on the unique history and the critical importance of context on the manifestations of the symptoms. Perhaps the present emphasis on discrete “autistic” behaviors tied to specific interventions should be seen in terms of more circumscribed value and utility.

## Developmental vs. acquired symptoms

In addition to the personalized nature of the characteristics and the dynamic nature of the manifestations of a movement difference mentioned above, it is impossible to overemphasize the importance of the developmental aspects of movement differences in autism vs. adult acquired disorders. For example, bradykinesia, or very slow movements, might have a wide range of effects on adults with acquired disorders such as Parkinsonism. In an infant or a toddler, the possible effects of slow responding or delayed initiating would surely have an effect on the entire trajectory of development even if the difference were intermittent or barely perceptible to the parents or professionals. Of course, we are not suggesting that these autistic people have Parkinson's syndrome; rather that they report sensory and movement differences which are not obvious to their caregivers, particularly parents of young children. Yet, the potential changes to the “dance of relationships” (Stern, 2000) alone would be worthy of many dissertations in child development. But the complexity of the task ought not deter us from attempting such inquiry because it could have enormous implications for our understanding of human development and diversity.

## Interpretation of symptoms as volitional

Many of us have accepted without question the implicit message that unusual movements presented by people with autism are always volitional and often pleasurable. Sensory and movement difference symptoms in autism are consistently interpreted by others as *autistic behaviors*. Neurological symptoms such as sudden, loud vocalizations; being in constant motion; extreme response to minor changes; unusual mannerisms and gait; and “unmotivated” laughter are examples of behaviors commonly thought to be performed “on purpose” and targeted for behavioral intervention. A social interpretation of these symptoms often leaves people with the assumption that they occur as a matter of choice, apathy, or learned behavior. Aggression during an episode of catatonic frenzy is viewed differently if the neurological aspects of the person's experience are considered. Typically, reprimands or contingent praise would not be used to change a recognized neurological symptom. As noted, the non-volitional aspects of behavior are rarely considered for people with autism. For example, the authors have all too often heard criticism and disparaging descriptions such as *lazy* or *non-compliant* applied to a person with autism who is in a non-responsive state. Frequently, the difficulty is related to stress, even the stress of excitement. An all too typical example is staff or family reporting that the child or adult *refused* to get out of the car or van to go to a place; he or she seems to like. Intervention or support that is based on our social interpretations of symptoms may not always be helpful. Returning the *non-compliant* person to home, school, or program usually results in additional trouble. We need a clearer understanding of people's experiences if we are to provide appropriate care and support that boosts self-confidence and is the product of collaboration rather than control. Donnellan et al. (2006) offer many suggestions for accommodations that may help people with autism deal with these situations.

## Interpretation of symptoms as meaningless

Our assumptions about a person's intention or meaning directly influence the way we respond moment to moment, the relationships we form and the support we give to people. When we label aspects of a person's behavior as meaningless, we may miss opportunities to extend learning and develop our relationships. Echolalia serves as well as an illustration. In the early years of behavioral intervention for people with autism (e.g., 1960–1980), professionals assessing a child's communication abilities were trained to assume that echolalia was the “meaningless repetition of a word or word group just spoken by another person” (Fay, 1969, p. 39), a non-functional, undesirable and “sick” behavior of autism (Lovaas, 1966; Lovaas et al., 1974), and a communication disorder in itself to be extinguished through behavior modification (Lovaas, 1977). The fine and detailed work of researchers such as Baltaxe and Simmons (1977), Prizant and Duchan (1981), and Prizant and Rydell (1984) began to influence our assumptions about the intentions of autistic speakers. Many people now understand that echolalia is neither always meaningless nor always meaningful. Although sometimes not intentional, many who lack other strategies for communicating may use echolalia intentionally to maintain relationships, improve their comprehension of spoken language and to express meaning (see Kanner, 1946). Acknowledgment of a person's efforts to accommodate, improvise, and create meaning is a cause for celebration and an opportunity to improve communication and boost self-esteem.

## Interpretation of symptoms as “not interested” in relating or communicating

People with autism often communicate, behave and participate in unique, very personal, perhaps idiosyncratic ways, that require their partners to be more flexible and open than usual in interpreting meaning and intention. Differences in the way people are able to use their bodies and focus their attention lead many to assume that a person does not care to participate or communicate and does not desire relationships. These assumptions affect our expectations, the way we speak with them and the educational and social opportunities we offer to them. Under the “criterion of the least dangerous assumption” (Donnellan, 1984) it is safest to assume that relationships are critical to human beings for learning and development even if, and perhaps especially if, they have difficulties in these areas (Fogel, 1993; Robledo and Donnellan, 2008).

## The critical importance of relationship in learning and development

The past 40 years have witnessed the growth of a body of knowledge, approaches, and intervention methodologies designed to address the needs of individuals with autism. Often the kinds of intervention strategies at our disposal are based on ideas and theories that conflict with each other. The content of interventions may be highly prescriptive or more loosely defined. Research can be cited in support of the efficacy of any kind of approach for at least some individuals in some situations. We struggle as well as to explain and describe that quality within any intervention that works and leads to growth and development between the partners involved. Perhaps, the essential factor underlying any successful intervention has been overlooked or at least not credited in the research. We propose, along with a growing number of investigators, that the undefined element is the presence and nature of the relationship between persons in any interaction (Maurer, 1993; Hill and Leary, 2009).

The role of relationship in learning is the centerpiece of socio-cultural psychology. While most of us believe that learning is enhanced by a facilitative relationship with a *more mature thinker*, western psychology has only recently directed attention to the nature of that relationship. Lev Vygotsky (1896–1934) was a Russian psychologist whose work described and defined the role of relationship in human development. His work emphasized the notion that *cognitive and specific skill development* is the result of *internalizing* interactions with others within a *relationship* (Bedrova and Leong, 1996). Ylvisker and Feeney (1998) have translated Vygotskian theory into a support model that focuses on apprenticeship and collaboration between the person and another with more expertise in the areas where support is needed. The “tutor” provides collaborative mediation that is fine-tuned to the learner's changing needs for support to enable participation in meaningful, project-oriented work: “The roots of cognitive, executive and communication functions, as well as behavioral self-regulation, are everyday social interaction routines” (Ylvisker and Feeney, 1998, p. 15–16). In the socio-cultural models of development, relationship with others serves as the springboard for learning. Learning happens within a social context, within a dialogue with others. We acquire cognitive skills, knowledge and *behavior regulation*, not simply through memorization of facts or actions, but through our interactions in the social world where this knowledge has function and meaning.

## Inconsistency in abilities

**Figure 1:**
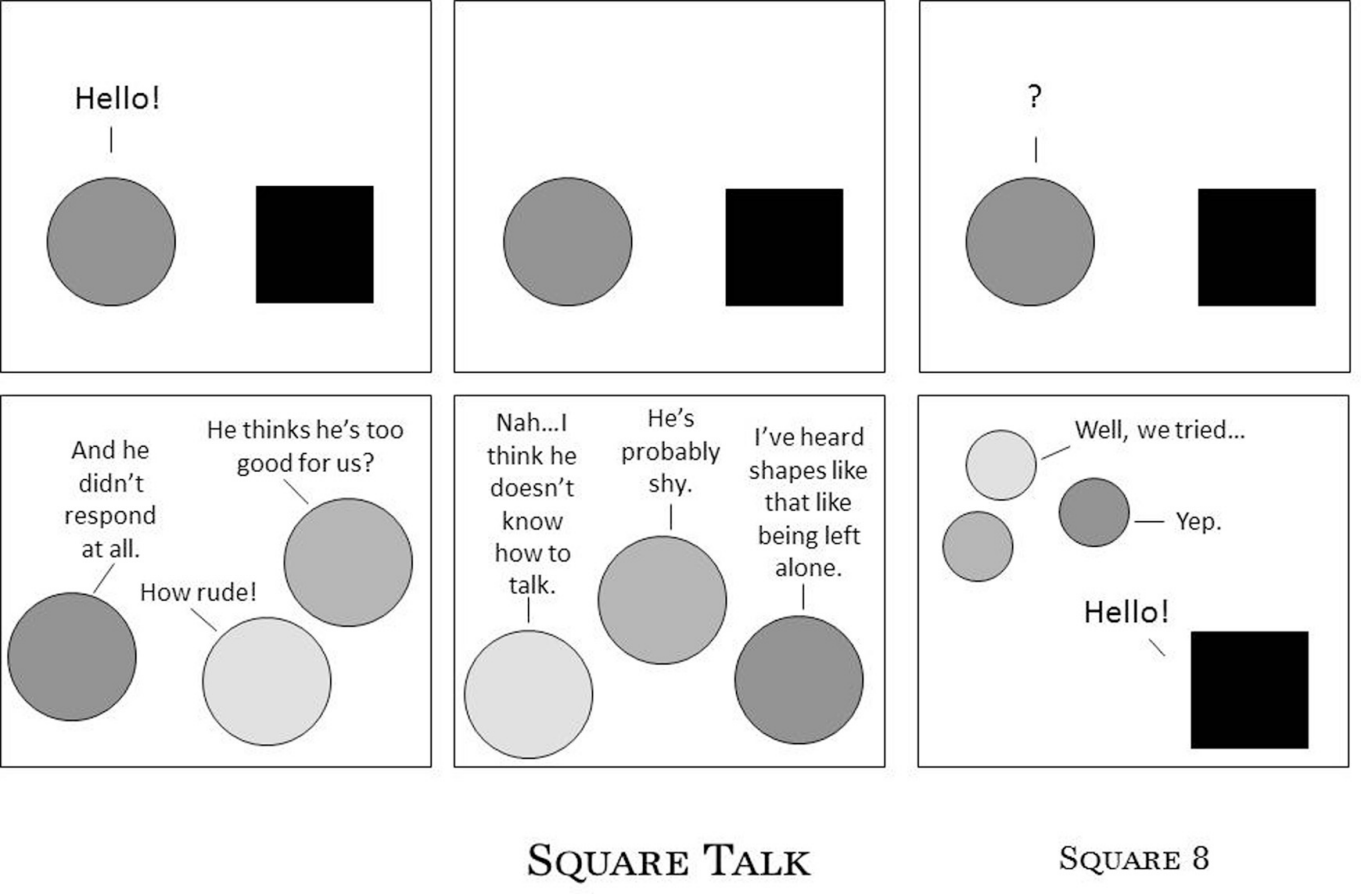
**Square Talk.** Harp (2008), reprinted with permission.

People report sensory and movement inconsistencies such as: fluctuations in speed and clarity of sensory perception; unreliable ability to maintain or release body postures; delays in speed and accuracy of movement and speech; unpredictable changes in muscle tone; and unwanted vocal, verbal, and physical tics and extraneous non-functional movement (e.g., Mirenda and Donnellan, 1986; Williams, 1996a; Harp, 2008; Robledo et al., 2012). A sensory and movement difference is characterized by this inconsistency, causing stress for the most common of movements (Baggs, 2007). A person struggling with these performance characteristics may not be able to predict, plan for or sustain effective participation. For example, a person with a 14-s delay in her ability to respond to others (e.g., Mirenda and Donnellan, 1986) is likely to be misinterpreted and misunderstood and unlikely to be offered time to respond. This is illustrated by Figure 1, Harp (2008) on her blog *Asperger's Square 8* (used with permission).

## Supporting self-esteem

Humans carry inside themselves an image that includes reasons for and the possibility of change. We need to know that we are OK just as we are, even though there are things we may want to learn or to do better.

A current trend in early intervention for young children with autism is to provide guidance in massive quantities (e.g., 40 h a week of one-to-one instruction). This guidance is naturally accompanied by frequent corrections and redirection. Given the intensity of this intervention, special care is needed to promote children's self-esteem at any age.

Equally important is the need for positive, optimistic, respectful support for adults with autism. The paucity of quality programs, diminished opportunity for interesting lives, effects of medication and chemical restraint are just a few of the additional burdens on these individuals and their families. Issues of collaboration, personalization, and comfort are also essential for children and particularly pressing for the adult population with the autism label. McGinnity and Negri (2005) offer helpful suggestions on how students and staff can learn to be more sensitive to the differences in those on the autism spectrum.

## Collaboration, personalization, and comfort

The growth of the autism industry over the past two decades has spawned no end of books, interventions, programs and products. Yet, the diagnosis of autism is not prescriptive of the type of supports needed for assisting any particular person to participate, relate, and communicate. Supports for people with autism should be personalized, reflect the respect and dignity due to all people and address the challenges with which people struggle to organize and regulate themselves in response to the sensory environment and their movement differences. Appropriate supports require a deep and local knowledge of the individual. This can be gained from those who know and appreciate them, but often such information is not available. Then it is even more essential to spend significant time with the person in a variety of activities and settings and with people who respect and admire him or her. We need to learn to listen with all of our senses and compassion (e.g., Lovett, 1996; Savarese, 2007) and to “presume competence” (Biklen and Cardinal, 1997) in all interactions. We do not put people in jeopardy by overestimating their experience. We do look for competence instead of deficits and talk to people in age-appropriate ways. And we model such interactions for all those who are, or may become, willing to know them better.

Moreover, we need to remember that in our journey of change, we all need allies who will collaborate with us to find the most comfortable and effective ways for us to learn to participate in our families, with our friends and as contributing members of our communities (Schwarz, 2004; Robledo and Donnellan, 2008). This is particularly critical for those persons who are challenged by the movement differences that often make such comfort temporary, personhood elusive, and collaboration a mystery. There is much to be learned from self-advocates with autism as well as from individuals who share some of the symptoms of movement differences such as Tourette syndrome, Parkinson's disorder and from their supporters (e.g., Williams, 1992; McGoon, 1994). For example, individuals with Tourette syndrome have taught us that calling attention to a behavior might make it much more difficult for a person to inhibit that behavior. It is roughly analogous to telling a stutterer not to stutter. Anyone familiar with classrooms and programs that have people with autism will recognize the value of that cautionary comment.

## Conclusion

When I was growing up, speaking was so frustrating. I could see the words in my brain, but then I realized that making my mouth move would get those letters to come alive, they died as soon as they were born. What made me feel angry was to know that I knew exactly what I was to say and my brain was retreating in defeat …

(Burke, 2005, p. 250)

Jamie Burke is a college student who now is able to speak the words he types with two fingers on his Augmentative and Alternative Communication (AAC) device. We have proposed that many other individuals with the autism label may be challenged by sensory movement differences in starting, stopping, executing, combining, and/or switching actions, thoughts, emotions and speech. These symptoms have been described in the literature for many years but generally not integrated into our descriptions or understanding of *autistic behaviors*.

Sensory and movement differences often escape the notice of those of us who do not typically experience them but have been well-described by autistic self-advocates and persons interested in individuals with autism and other disability labels. Ignoring these differences (or redefining them as *autistic behaviors* to be controlled) has made life unnecessarily more difficult for individuals with autism and those who care about and for them. Many of the assumptive errors we have made are based on our own social history. In the absence of clarity about the nature of these movement differences, we will continue to be forced into the default position of seeing all unfamiliar behaviors as intentional, deliberate evidence of intellectual impairments and even pleasurable. We have not proposed another list of deficits but a greater understanding of the complexity of what we call *autistic behaviors* and the necessity to rethink our assumptions about them. The task is not going to be easy. Such sensory movement differences are manifest in autism and many other disorders in strikingly unique, personalized and dynamic ways that test present research strategies that rely heavily on a positivist-reductionist philosophy. Yet, some of the brightest scientific lights of the twentieth century reminded us that the best way to approach objectivity in science is to view the phenomenon from as many perspectives as possible (Luria, 1932; Edelman, 1992; Arthur Schawlow, pers. communication, 1996). As Einstein shared: “Not everything that counts can be counted and not everything that is counted, counts” (Einstein, 2004 as quoted in Cunningham and Scott, 2004, p. 208).

There is a long, continual path of misunderstanding in the field of autism. People have been thought of, and referred to, as “non-persons,” “behavior problems,” and “sub-normal” in every imaginable way. If they cannot speak, we assume they have little to say and offer only the most limited of communication options. Irrespective of the precision and intensity of our interventions, more often than not they experience isolation, segregation, homogeneous grouping, loneliness, pain, and boredom as part of their customary care across the life span. Often their sensory and movement differences contribute to such outcomes as these leave the rest of us unaware of the true nature of their challenges.

Any view of autism at this time needs to reflect the experience of self-advocates with autism and others who describe sensory and movement differences, as well as the latest in the neuroscience and child development literature. We need a research agenda that focuses on understanding and supporting autistic people and others in more respectful, personalized, and successful ways. It is the least dangerous assumption (Donnellan, 1984) to see all as full human beings who may have formidable and unfamiliar challenges to overcome and who, of course, desire social interaction, communication and participation.

Too often autistic children are raised to believe they are broken and need to be fixed. Adults with autism too often live lives of isolation and poverty. Understanding people's experiences may lead to acceptance, accommodation and appropriate support. To continue down the same paths, well-worn for 65 years, when all these data impel us to *re*think our assumptions and broaden our path is *un*thinkable.

### Conflict of interest statement

The authors declare that the research was conducted in the absence of any commercial or financial relationships that could be construed as a potential conflict of interest.

## Footnotes

^1^For reviews of the complex and dynamic interrelationship of movement, perception, relationship, and cognitive development, see: (Gibson, 1979; Thelen and Smith, 1994; Stern, 2000).
